# The role of mitophagy in metabolic diseases and its exercise intervention

**DOI:** 10.3389/fphys.2024.1339128

**Published:** 2024-01-29

**Authors:** Shaokai Tang, Yuanwen Geng, Qinqin Lin

**Affiliations:** School of Physical Education, Yanshan University, Qinhuangdao, China

**Keywords:** mitophagy, exercise, metabolic disease, mitochondrial dysfunction, exerkine

## Abstract

Mitochondria are energy factories that sustain life activities in the body, and their dysfunction can cause various metabolic diseases that threaten human health. Mitophagy, an essential intracellular mitochondrial quality control mechanism, can maintain cellular and metabolic homeostasis by removing damaged mitochondria and participating in developing metabolic diseases. Research has confirmed that exercise can regulate mitophagy levels, thereby exerting protective metabolic effects in metabolic diseases. This article reviews the role of mitophagy in metabolic diseases, the effects of exercise on mitophagy, and the potential mechanisms of exercise-regulated mitophagy intervention in metabolic diseases, providing new insights for future basic and clinical research on exercise interventions to prevent and treat metabolic diseases.

## 1 Introduction

Life-sustaining activities are dependent on metabolism, and disturbances in metabolic functions (such as the metabolism of lipids, amino acids, glucose, proteins, electrolytes, and energy) can damage one or multiple organs (Kaelin and McKnight, 2013; Green et al., 2021), ultimately leading to various metabolic disorders, including obesity, diabetes mellitus, and nonalcoholic fatty liver disease (NAFLD) (Lee and Olefsky, 2021; Jabbour and Taheri, 2022; Chaudhry et al., 2023), and development of several associated complications. Together, these result in the formation of metabolic syndrome (MS) in severe cases (Castro-Barquero et al., 2020). MS is a chronic disease characterized by obesity and insulin resistance, which can further increase the risk of cardiovascular disease (CVD) and type 2 diabetes mellitus (T2DM) (Heindel et al., 2017). Neurodegenerative diseases (NDDs) are not explicitly categorized as metabolic diseases but owing to the similarity in pathogenesis, they are considered among complications triggered by metabolic dysfunction. The pathogenesis of metabolic diseases has not yet been established. However, they mainly occur due to the surge in reactive oxygen species (ROS) that triggers mitochondrial dysfunction, DNA damage, and protein structural disorders, ultimately leading to oxidative stress (OS) and inflammation, and exacerbating the development of a cascade of metabolic diseases (Urman et al., 2019; Silveira et al., 2022). In recent years, the incidence rate of metabolic diseases has been increasing yearly, seriously affecting human health and quality of life (Jiang et al., 2023). Currently, the use of drug substitution therapy is the mainstream method for the treatment of metabolic diseases. However, problems such as high drug dependence, complex adverse reactions, poor efficacy, and inability to cure remain prominent (Qindeel et al., 2020). Therefore, in-depth exploration of potential targets and alternative methods for preventing and treating metabolic diseases has important theoretical significance and clinical guidance value.

Autophagy is a highly conserved self-protective mechanism that maintains cellular and metabolic homeostasis by engulfing damaged and senescent organelles or pathogens and encapsulating them into vesicles, fusing them with lysosomes to form autophagic lysosomes, and degrading, recycling, and reusing their internal metabolites (Galluzzi et al., 2017; Dikic and Elazar, 2018). According to the different degradation mechanisms, autophagy can be divided into three types: macroautophagy, microautophagy, and molecular chaperone-mediated autophagy (Andrade-Tomaz et al., 2020; Ma S. et al., 2022). Mitochondrial autophagy (Mitophagy), which belongs to one of the types of macroautophagy and was first proposed by Lemasters in 2005, is an intracellular mechanism of selective degradation targeting dysfunctional mitochondria. It plays an essential role in the maintenance of homeostasis of the intracellular mitochondrial quality control (MQC) system and the regulation of mitochondrial energy metabolism (Lemasters, 2005; Ajoolabady et al., 2022). However, when mitochondria are damaged under stress (such as hypoxia and accumulation of misfolded proteins), the mitochondrial permeability transition pore opens, leading to mitochondrial depolarization and inducing the activation of mitophagy-associated proteins. Damaged mitochondria are subsequently enclosed specifically by the autophagosome, and transferred to the lysosome, where it fuses with the lysosome to form mature mitochondrial autolysosomes. Finally, damaged mitochondria are degraded by lysosomes (Lu et al., 2023). The role of mitophagy in metabolic diseases has been extensively studied in recent years. Optineurin (OPTN)-mediated mitophagy is over-activated in the lung tissues of septic acutely lung-injured mice with cecal ligation-perforation (CLP) constructs, and its inhibition attenuates CLP-induced acute lung injury (Ling et al., 2023). Inhibition of Parkin- and PTEN-induced putative kinase 1 (PINK1)-mediated mitophagy exacerbates hepatic lipogenesis, inflammation, and insulin resistance in NAFLD mice, and upregulation of mitophagy levels effectively ameliorates hepatic steatosis in NAFLD mice (Chen et al., 2023). Mitophagy is a “double-edged sword” in the development of metabolic diseases, and targeted improvement of mitophagy abnormalities can serve as a new strategy for the prevention and treatment of metabolic diseases. In addition, exercise intervention, an essential supplement to drug therapy, can regulate mitophagy levels and exert metabolic protective effects. Wheel running exercise for 9 weeks significantly upregulated the microtubule-associated protein 1 light chain 3 (LC3)-II/LC3-I ratio in brain tissue of elderly cerebral ischemia-reperfusion (CI/R) injury mice established by middle cerebral artery occlusion (MCAO) surgery and increasing the levels of LC3-II, p62, and autophagy associated protein 7 (ATG7). The expression of Bcl-2 adenovirus/E1B19 kDa interacting protein 3 (BNIP3), Parkin, mitofusin 2 (Mfn2), and mitochondrial-related protein 1 (Drp1) promotes mitophagy and mitochondrial fusion/fission, thereby improving behavioral cognitive ability and neural damage in elderly CI/R injury mice (Qin et al., 2023). This review comprehensively summarizes the role of mitophagy in metabolic diseases, the effects of different types of exercise on mitophagy, and the potential mechanisms of exercise-regulated mitophagy intervention in metabolic diseases, providing new insights for basic and clinical research on exercise intervention for preventing and treating metabolic diseases.

## 2 MQC balance

**FIGURE 1:**
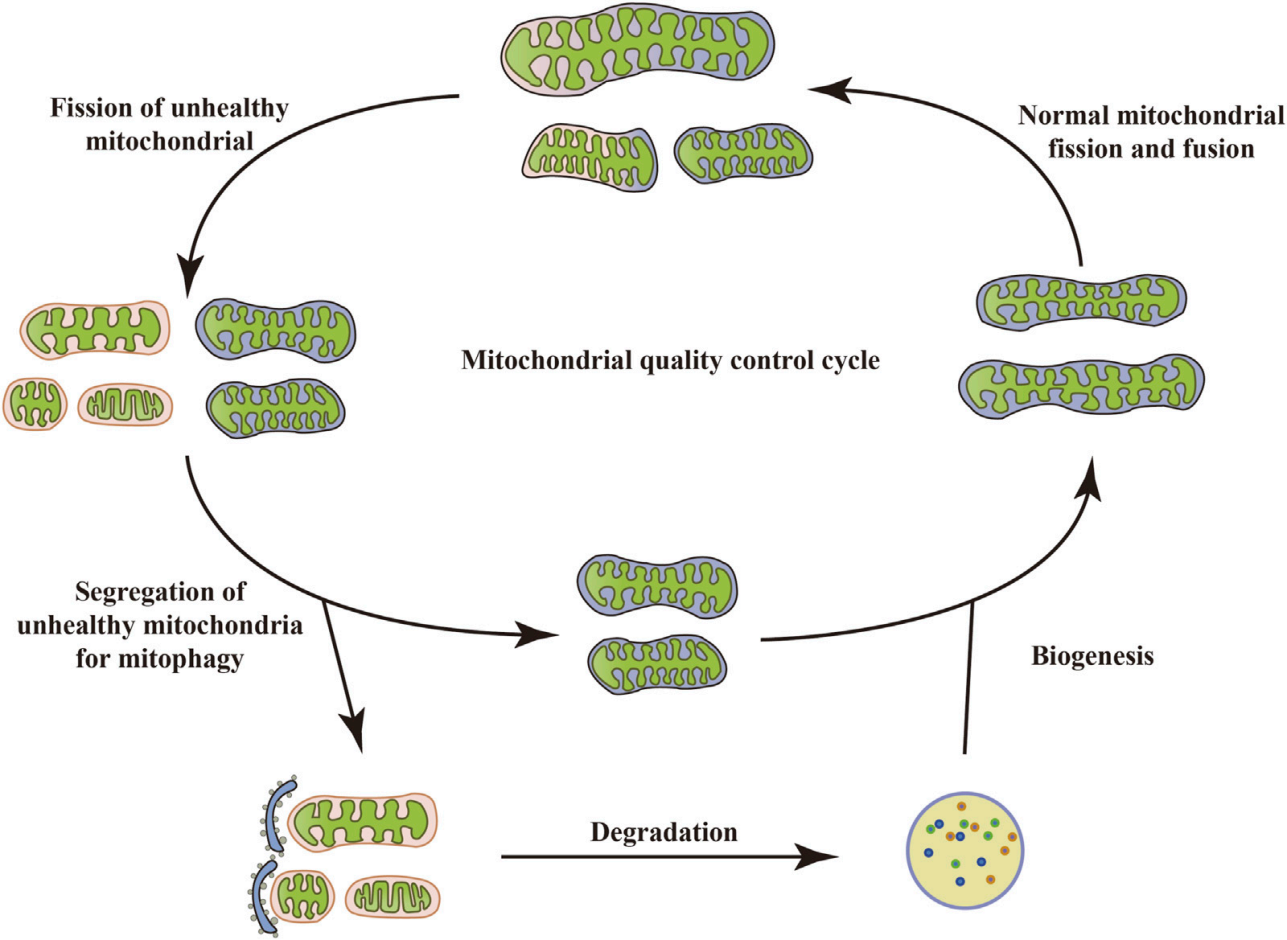
Mitochondrial quality control cycle. When mitochondria become depolarized or dysfunctional, they are labeled as “abnormal” and degraded. Once the abnormal mitochondria are labeled, the unhealthy mitochondria undergo fission with the intact mitochondrial network. Some damaged mitochondria also fuse with other healthy mitochondria around them in an attempt to restore their normal physiological function. However, typically, dysfunctional mitochondria undergo mitophagy, which in turn recycles and degrades the mitochondria. Upon completion of degradation, cells recycle amino acids and FAs, allowing the remaining healthy mitochondrial network to grow and undergo fission through biogenesis, thus maintaining homeostasis of the cellular environment.

Mitochondria are specialized double-membrane intracellular compartments or organelles that exist in various cells, commonly referred to as the “energy factory of cells.” They are the leading site for oxidative phosphorylation of cells to synthesize adenosine triphosphate (ATP) (Chaban et al., 2014; Yao et al., 2021; Terešak et al., 2022). Mitochondria, as determinants of metabolic health, are critical for several physiological processes such as inflammatory responses, OS, immune signal transduction, apoptosis, and stress response (Harrington et al., 2023). Mitochondrial damage and dysfunction are mainly manifested as abnormal ATP supply, increased ROS production, structural changes in mitochondrial cristae, and opening of mitochondrial membrane permeability transition pores, which are key factors leading to the occurrence and development of many metabolic diseases (Zhou T. et al., 2023). To maintain mitochondrial quantity and physiological function, cells can initiate a series of protective mechanisms under stress, including mitochondrial biogenesis, mitochondrial fission/fusion, mitophagy, mitochondrial transport, and others. This is called MQC. Typically, mitochondria exist mainly in a highly dynamic network of mitochondrial interconnections, and this dynamic characterization is a result of the constant interweaving of MQC cycles (Held and Houtkooper, 2015). When depolarized or dysfunctional mitochondria become “abnormal”, they are degraded. Once the abnormal mitochondria are labeled, fission of unhealthy mitochondria with the intact mitochondrial network occurs. Certain damaged mitochondria fuse with other healthy mitochondria around them in an attempt to restore their normal physiological function (Chan, 2006). However, typically, dysfunctional mitochondria undergo mitophagy. When dysfunctional mitochondria are separated from the healthy mitochondrial network, the damaged mitochondria are first split into fragments, which are quickly wrapped by autophagosomes and then enter the autophagosome through specific wrapping. This leads to the formation of double-membrane autophagosomes that phagocytose the mitochondria and fuse with lysosomes, facilitating the recycling and degradation of mitochondria. Upon completion of degradation, cells recycle amino acids and fatty acids (FAs), allowing the remaining healthy mitochondrial network to grow and undergo fission through biogenesis (Kim et al., 2007), thus maintaining homeostasis of the cellular environment (Figure 1). However, excessive/insufficient mitophagy, inhibition of mitochondrial biogenesis, and an overall reduction in protein synthesis can lead to mitochondrial dysfunction, causing insufficient ATP production or an increased burden of ROS, which in turn cannot meet the cellular demands, ultimately leading to energy deprivation and cell death. Thus, regulation of mitophagy may be a valuable therapeutic option that targets the elimination of dysfunctional mitochondria while still providing sufficient energy to mitigate cellular damage, restore protein translation, and ultimately restore homeostasis (Anzell et al., 2018).

## 3 Overview of mitophagy

**FIGURE 2:**
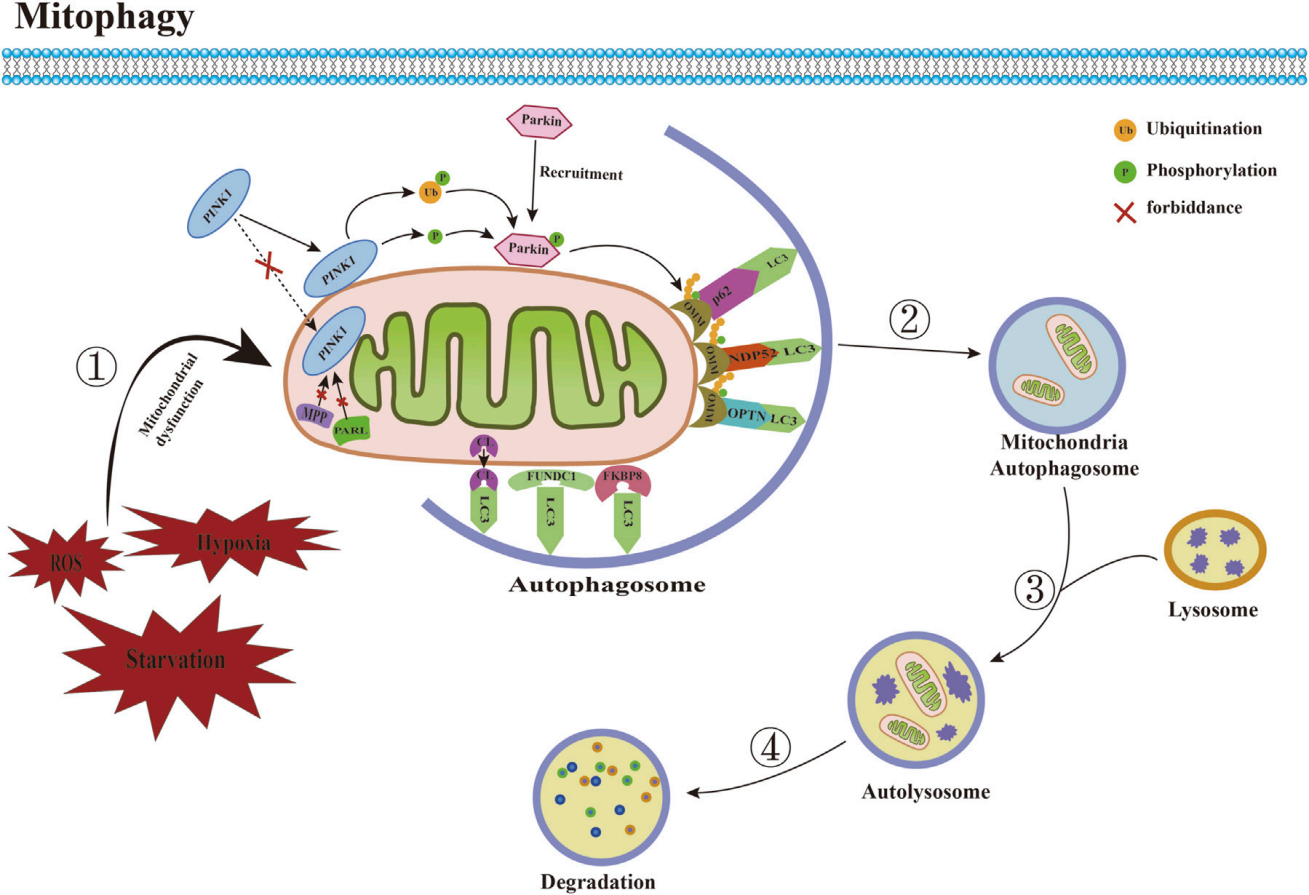
Committed step of mitophagy. ① Under conditions such as ROS accumulation and hypoxia, mitochondrial dysfunction occurs. ② Mitochondria are engulfed by autophagosomes to form mitochondrial autophagosomes. ③ Mitochondrial autophagosomes fuse with lysosomes to form mature mitochondrial autolysosomes. ④ The contents of mitochondria are degraded by lysosomes. Abbreviations: ROS, reactive oxygen species; LC3, microtubule-associated protein 1 light chain 3; PINK1, PTEN induced putative kinase 1; MPP, mitochondrial-processing protease; PARL, Presenilin-associated rhomboid like; CL, Cardiolipin; FUNDC1, FUN14 domain-containing protein 1; FKBP8, FK506 binding protein 8; OMM, outer mitochondrial membrane; NDP52, nuclear do-main 10 protein 52; OPTN, optineurin.

Mitophagy is a mitochondria-specific form of autophagy that selectively degrades damaged mitochondria through receptors (Figure 2). Currently, many studies have confirmed that a variety of proteins and pathways mediate the process of mitophagy. According to the different recognition mechanisms between phagocytosed vesicles and damaged mitochondria, they can be roughly divided into two pathways: ubiquitin-dependent and receptor-dependent pathways. 1) The ubiquitin-dependent pathway refers to the extensive ubiquitination of proteins that depend on the surface of the mitochondrion to promote mitophagy. The PINK1/Parkin is a critical mediator in the classical mitophagy pathway and has been extensively studied (Han H. et al., 2023; Xia et al., 2023). Under physiological conditions, cytoplasmically synthesized PINK1 first interacts with the translocase of the outer membrane (TOM) to enter the mitochondrial membrane gap (Kato et al., 2013). It is then translocated to the mitochondrial inner membrane by translocase of inner membrane (TIM) (Sekine et al., 2019), and then cleaved by the mitochondrial inner membrane protein presenilin-associated rhomboid-like (PARL) and mitochondrial matrix protein mitochondrial-processing protease (MPP) shear PINK1 (Greene et al., 2012; Meissner et al., 2015), which translocates from the inner mitochondrial membrane to the cytoplasm. Ultimately, it is degraded by cytoplasmic ubiquitin protein ligase E3 component N-recognin 1 (UBR1), UBR2, and UBR4 through the proteasome pathway, during which Parkin E3 ubiquitin ligases located in the cytoplasm are usually inactive and exist in their natural autoinhibited conformation (Yamano and Youle, 2013). When mitochondrial depolarization damage occurs, the mitochondrial membrane potential (MMP) decreases, and PINK1 hydrolysis is inhibited, which stably binds to the damaged mitochondrial outer membrane, recruiting Parkin to translocate from the cytoplasm to the mitochondrial outer membrane, thereby phosphorylating and activating Parkin activity (Fiesel et al., 2023). Activated Parkin binds damaged mitochondria and ubiquitinates different mitochondrial outer membrane proteins to interact with several junction proteins containing ubiquitination binding sites and LC3-interacting region (LIR), including OPTN, nuclear do-main 10 protein 52 (NDP52), and sequesto-some 1 (SQSTM1)/p62. Moreover, it is recognized by LC3 through the LIR motif, which then transports dysfunctional mitochondria into autophagosomes, where degradation ultimately occurs in autolysosomes to complete mitophagy (Uoselis et al., 2023). 2) The receptor-dependent pathway refers to the direct binding of the LIR motif mitophagy-related receptor to LC3, independent of ubiquitination, and the transport of damaged or redundant mitochondria to autophagosomes to achieve mitochondrial selective autophagic degradation. Studies have demonstrated that various types of mitophagy-related receptors in mammalian cells can mediate this process. Mitochondrial swelling, vesicle formation, and somatic cristae breakage were observed in the CuSO4-induced kidney injury model, nip3-like protein (NIX)/BNIP3 mRNA and protein levels were significantly upregulated, and the number of mitochondrial and lysosomal fluorescent aggregation sites was increased, suggesting that CuSO4 treatment promotes the onset of BNIP3/NIX-mediated mitophagy, and that co-treatment with CuSO4 and an inhibitor of mitophagy significantly exacerbates mitochondrial dysfunction (Bai et al., 2023). Focused low-intensity pulsed ultrasound (FLIPUS) treatment significantly enhanced the protein level of phosphoglycerate mutase 5 (PGAM5) in interleukin-1β (IL-1β)-induced primary mouse chondrocytes of knee joints, promoted Ser13 dephosphorylation containing FUN14 do domain protein 1 (FUNDC1), activated mitophagy in chondrocytes, significantly upregulated the expression of collagen II (Col II) and B-cell lymphoma/leukemia-2 (Bcl-2), downregulated matrix metalloproteinase 13 (MMP13), Bcl-2 associated X protein (Bax), and cleaved caspase-3 expression, and attenuated extracellular matrix (ECM) degradation and apoptosis. *In vivo* experiments further confirmed that FLIPUS could alleviate ECM loss and chondrocyte apoptosis in a destabilization of the medial meniscus (DMM) mouse model through mitophagy mediated by FUNDC1, thereby exerting a protective effect on cartilage (Ye et al., 2023). In addition, syntaxin 17 (STX17) (Xu et al., 2023), FK506 binding protein 8 (FKBP8) (Yoo et al., 2020), nucleotide-binding domain and leucine-rich repeat-containing family member X1 (NLRX1) (Zhang Y. et al., 2019), prohibitin 2 (PHB2) (Lai et al., 2023), cardiolipin (CL) (Yang et al., 2023), thioredoxin-interacting protein (Txnip) (Zhang S. et al., 2023), dual-specificity protein phosphatase 1 (DUSP1) (Li R. et al., 2023), and other mitophagy-related receptors have also been shown to mediate mitophagy under stress conditions.

## 4 Mitophagy and metabolic diseases

The specific pathogenesis of metabolic diseases has not been conclusively determined, and it is currently believed that potential mechanisms such as ROS surge, abnormal protein structure and function, chronic low-grade inflammation, DNA damage, and insulin resistance are closely related to mitochondrial dysfunction (Matsumura et al., 2023). It has been found that mitochondrial damage or dysfunction can contribute to ROS accumulation and cell death, leading to dysregulation of energy metabolism and cellular dysfunction, which subsequently induces inflammation and OS. This forms a “vicious circle” and ultimately leads to metabolic disorders (Amorim et al., 2022; Fahed et al., 2022; Kuznetsov et al., 2022). Many studies have confirmed that abnormal mitophagy is a key factor in the occurrence and development of metabolic diseases, and targeted improvement of mitophagy abnormalities may become a new entry point for future prevention and treatment of metabolic diseases (Table 1).

**TABLE 1:** The roles of mitophagy in metabolic diseases.

Metabolic diseases	Compounds/Drugs/Proteins/RNA	Mitophagy	Marker proteins	Main functions	Authors
obesity	DHA ↑	↑	PPARγ, LC3, BNIP3, NIX1 and NIX2 ↑	Promoted adipocyte apoptosis and reduced abdominal fat content in grass carp	Bian et al. (2023)
obesity	NR1D1 ↑	↑	ULK1, Parkin and PINK1 ↑	reduced lipid droplet production and attenuated obesity	Yu et al. (2022)
			TOM20 ↓		
obesity	miquelianin-rich LLE ↑	↓	p62 and Drp1 ↑	promoted beige adipocyte formation and enhanced energy expenditure	Wang Z et al. (2023)
			PINK1, Beclin1, LC3B and parkin↓		
obesity	SEM ↑	↓	p62 ↑	promoted browning of white fats and inhibited lipid accumulation	Lin et al. (2021)
			PINK1and Parkin ↓		
T2DM	SEND ↑	↑	Parkin, PINK1, and LC3-II/I ↑	inhibited ERS and apoptosis and improved pancreatic β-cell function	Huang et al. (2023)
T2DM	Silibinin ↑	↑	PINK1 and Parkin ↑	inhibited iron death and improved glucose and FA metabolism in INS-1 cells	Du et al. (2023)
T2DM	CK ↑	↑	PINK1, Parkin, MFN1, MFN2, and OPA1 ↑	improved the mitochondrial quality and function and alleviated insulin resistance	Li W et al. (2023)
			FIS1 and Drp1 ↓		
T2DM	C3G ↑	↑	PINK1 and Parkin ↑	alleviated the islet OS induced by hyperglycemia in diabetic mice, and ameliorated T2DM	Ye X et al. (2021)
NAFLD	CP ↑	↑	PGC-1α, PPARα, PINK1	inhibited FFA-induced OS and lipid accumulation	Yao Z et al. (2023)
			Parkin, ATG7 and LC3I/II ↑		
			P62 ↓		
NAFLD	GDNF ↑	↑	SIRT3, PINK1 and OPA1 ↑	Inhibited OS in hepatocyte	Mwangi et al. (2022)
NAFLD	ZNF143 ↓	↑	PINK1, Parkin, Beclin-1 and LC3-II/I ↑	Mitigated hepatic steatosis in hepatocyte	Dong et al. (2023)
NAFLD	Que ↑	↑	ATG5, ATG12, LC3, PINK1 and Parkin ↑	Inhibited inflammatory response and OS, alleviated hepatic atrophy and lipid accumulation in NAFLD mice	Cao P et al. (2023)
HF	Ginsenoside Rg3 ↑	↑	ULK1 and FUNDC1 ↑	Inhibited OS and inflammatory response, improved cardiac function in HF rats	Wang X et al. (2023)
AS	XMK ↑	↑	PINK1, Parkin, LC3, LAMP1 and TOMM20 ↑	Reduced plaque, lipids, and ROS in AS mice, prevented and treating AS	Cao Y et al. (2023)
			SQSTM1 ↓		
Myocardial Hypertrophy	Metformin and PINK1/Mfn2 ↑	↑	Beclin1and LC3 ↑ p62 ↓	Reduced ROS and apoptosis, improved myocardial hypertrophy and fibrosis in TAC mice	Ma Z et al. (2023)
Myocardial ischemia-reperfusion injury	miR-494-3p ↑	↓	Mfn1, Mfn2 and OPA1 ↑	Alleviated apoptosis and improved myocardial cell damage	Mu et al. (2023)
			LC3, Beclin1, PINK1 and Parkin ↓		
DKD	PAA ↑	↑	LC3, ATG5, p62 and FUNDC1 ↓	Inhibited podocyte apoptosis and inflammatory response, improved renal dysfunction in DKD mice	Wu et al. (2023)
Diabetic cerebral ischemia-reperfusion injury	Metformin ↑	↑	PINK1, Parkin, p62 and LC3-II/LC3-I ↑	Prevented excessive mitochondrial fission and apoptosis, reducing CI/R nerve damage	Guo et al. (2023)
			Drp1 and Cyt-c ↓		
DCM	L-carnitine ↑	↑	PINK1, Parkin and LC3B II ↑	Inhibited lipid peroxidation and enhanced endothelial barrier function, maintained microvascular structure in DCM mice	Li S et al. (2023)
Diabetic muscular dystrophy	Bavachin and Corylifol A ↑	↑	LC3-II, p62, Parkin	Inhibited OS and enhanced the muscle mass of diabetic muscular dystrophy mice	Yeon et al. (2023)
			PINK1 and BNIP3 ↑		
PD	Morin ↑	↑	PINK1, Parkin, LC3-II	Alleviated the loss of dopaminergic neurons and improved the behavioral defects of PD mice	Wang Z et al. (2023)
			AMPK and ULK2 ↑		
			Mfn1 and TOMM20↓		
AD	Loganin ↑	↓	Mfn2 and OPA1 ↑	Alleviated mitophagy dysfunction, restored learning and memory abilities in 3×Tg-AD mice	Zhou Y et al. (2023)
			Drp1, Fis1, LC3II, p62, PINK1, Parkin and TOMM20 ↓		
TNS	Melatonin ↑	↓	Tom20 and COXIV ↑	inhibited apoptosis in PC12 cells and HT22 cells	Zeng et al. (2022)
			PINK1 and Parkin ↓		
Alzheimer disease	PSS ↑	↑	PINK1, Parkin and LC3-II ↑	Inhibited NLRP3 inflammasome activation, improved cognitive function in AD mice	Qiu et al. (2022)
Parkinson’s disease	Circ EPS15 ↑	↑	PINK1, LC3 and TOMM20 ↑	Promoted the recovery of dopaminergic neurons in PD mice	Zhou YZ et al. (2023)
			SQSTM1/p62 ↓		

Abbreviations: DHA, docosahexaenoic acid; PPARγ, peroxisome proliferator-activated receptor γ; LC3, microtubule-associated protein 1 light chain 3; BNIP3, Bcl-2 adenovirus/E1B 19k D interacting protein 3; NIX, nip3-like protein; NR1D1, nuclear receptor subfamily 1 group D member 1; ULK1, UNC-51-like kinase 1; PINK1, PTEN induced putative kinase 1; TOMM20, translocase of the outer mitochondrial membrane member 20; LLE, lotus leaf extract; Drp1, dynamin-related protein 1; Beclin1, B-cell lymphoma-2-interacting myosin-like coiling-coil protein 1; T2DM, type 2 diabetes mellitus; SEND, selenium nanodot; ERS, endoplasmic reticulum stress; FAs, ;fatty acids; CK, compound K; Mfn2, mitofusin 2; OPA1, optic atrophy 1; Fis1, fission mitochondrial 1; C3G, cyanidin-3-O-glucoside; OS, oxidative stress; NAFLD, nonalcoholic fatty liver disease; CP, corn peptide; PGC-1α, peroxisome proliferator-activated receptor γ coactivator 1α; ATG7, autophagy associated protein 7; FFA, free fatty acid; GDNF, glail cell line-derived neurotrophic factor; SIRT3, silent information regulator 3; ZNF143, zinc finger protein 143; Que, quercetin; HF, Heart failure; FUNDC1, FUN14 do-main-containing protein 1; AS, atherosclerosis; XMK, xinmaikang; LAMP1, lysosome-associated membrane protein 1; SQSTM1/p62, sequesto-some 1; ROS, reactive oxygen species; TAC, transverse aortic constriction; DKD, diabetic kidney disease; PAA, Poricoic acid A; Cyt-c, Cytochrome c; CI/R, Cerebral ischemia/reperfusion; DCM, diabetic cardiomyopathy; PD, parkinson's disease; AMPK, AMP-activated protein kinase; AD, alzheimer disease; TNS, transient neurologic syndrome; PSS, polygala saponins; NLRP3, nucleotide-binding oligomerization domain-like receptor protein 3; ↑, up-regulated; ↓, down-regulated.

### 4.1 Mitophagy and obesity

Obesity is a typical metabolic disease characterized by expansion and abnormal accumulation of adipocytes (Lorente-Cebrián et al., 2019). In obesity, the mitochondrial function in the adipose tissue is abnormal, which contributes to disorders of adipose differentiation and production, impaired lipid metabolism, excessive inflammation, and insulin resistance, in turn inducing the exacerbation of the obese state (Wu et al., 2019). Promoting mitophagy inhibits lipogenesis and reduces obesity. Docosahexaenoic acid (DHA), a typical ω-3 polyunsaturated FA, is highly enriched in oils from deep-sea fish and marine microalgae and promotes adipocyte apoptosis to combat obesity in mammals and fish (Tatsumi et al., 2022). DHA significantly upregulates the mRNA expressions of BNIP3, NIX1, and NIX2 and promotes mitophagy in the abdominal adipose tissue of grass carp (*Ctenopharyngodon idellus*), in turn enhancing the mRNA expressions of caspase9, caspase3a, caspase3b, and Bax, and Bax/Bcl-2 ratio, and down-regulating that of Bcl-2. It increases the level of adipocyte apoptosis and reduces the abdominal fat content in grass carp. *In vitro* experiments have further confirmed that silencing of peroxisome proliferator-activated receptor γ (PPARγ) in primary adipocytes of grass carp significantly reduces the expression of BNIP3 and NIX, and decreases mitochondrial and lysosome co-localization, while preventing mitophagy-mediated adipocyte apoptosis, thus attenuating the inhibitory effect of DHA on lipid accumulation in adipocytes. DHA treatment significantly increases the expression of PPARγ in the primary adipocytes of grass carp. PPARγ binds to the LC3 promoter, thus increasing its expression, activates BNIP3-mediated mitophagy, induces apoptosis, and inhibits the accumulation of fat (Bian et al., 2023). The expressions of nuclear receptor subfamily 1 group D member 1 (NR1D1) and UNC-51-like kinase 1 (ULK1) are significantly reduced in an *in vitro* obesity model established using mouse preadipocyte (3T3-L1) differentiation, and increased NR1D1 could rapidly bind to the ULK1 promoter, thus significantly up-regulating the expression of ULK1, Parkin and PINK1 and down-regulating that of TOM20, increasing mitophagy levels, enhancing 3T3-L1 cell viability and triglyceride (TG) content, reducing lipid droplet production, and attenuating obesity. On the contrary, the knockdown of ULK1 reverses the benefits of overexpression of NR1D1 on obesity (Yu et al., 2022).

Another study confirmed that inhibition of mitophagy promotes the browning of white adipocytes and inhibits lipid accumulation. Lotus leaf is a medicinal and edible plant rich in several bioactive components, including miquelianin, which promotes weight loss and exhibits anti-inflammatory and antiviral properties (Yanan et al., 2023). mRNA expressions of Pink1, Parkin, B-cell lymphoma-2-interacting myosin-like coiling-coil protein 1 (Beclin1), and LC3B are significantly upregulated, and p62 expression was downregulated in an *in vitro* model of mouse embryonic fibroblast (3T3-L1) differentiation. This induces excessive mitophagy and prevents the beige coloration of 3T3-L1 cells. Miquelianin-rich lotus leaf extract (LLE) significantly increases the expression of β3-adrenergic receptor (β3-AR) and p-AMP-activated protein kinase (AMPK)α/AMPKα ratio in the inguinal white adipose tissues of *in vitro* models established by the differentiation of 3T3-L1 cells and in high-fat diet-induced obese mice, thus significantly decreasing the protein expression of the mitophagy markers, PINK1, Parkin, Beclin1 and LC3B, up-regulating the expressions of p62 and Drp1, increasing the expression of beige fat-enriched marker proteins nuclear respiratory factor 1 (Nrf1), mitochondrial transcription factor A (TFAM), PPARα, and cytochrome oxidase subunit VII a (Cox7a), and upregulated the protein levels of mitochondrial biogenesis markers, silent information regulator 1 (SIRT1), peroxisome proliferator-activated receptor γ coactivator 1α (PGC-1α), uncoupling protein 1 (UCP1) and fibroblast growth factor 21 (FGF21) levels. This suggests that LLE can inhibit the level of mitophagy and promote adipocyte beige coloration and mitochondrial biogenesis. Chemical inhibition shows that AMPK inhibitors significantly suppress the downregulation of mitophagy-associated proteins and the upregulation of DRP1 and beige fat-enriched markers in white adipose tissues of obese mice caused by LLE. Agonists enhance the changes in these indexes, suggesting that LLE may promote beige adipocyte formation through activation of the AMPK/DRP1/PINK1/Parkin signaling pathway to enhance energy expenditure (Wang Z. et al., 2023). Sesamol (SEM) is a small molecule natural lignan with a phenolic ring structure extracted from sesame seeds with a wide range of pharmacological effects (Jiang Y. et al., 2023). SEM significantly upregulates the protein levels of UCP1 in the white adipose tissue of high-fat diet-induced obese mice, downregulates serum TG and total cholesterol (TC) levels, promotes the browning of white adipose in obese mice, inhibits lipid accumulation, and improves high-fat diet-induced obesity. Cellular experiments show that SEM treatment significantly increases the expressions of β3-AR and protein kinase A (PKA) in mature 3T3-L1 cells, followed by upregulation of mitochondrial biogenesis markers, PGC-1α, NRF1, and TFAM, a significant reduction in mitophagy-associated markers, PINK1 and Parkin, increase the protein level of p62, and upregulate mRNA and protein expressions of Ucp1, suggesting that SEM can induce mitochondrial biogenesis, inhibit mitophagy, and promote browning of white adipocytes by activating the β3-AR/PKA pathway (Lin et al., 2021).

In summary, regulating mitophagy can significantly inhibit adipocyte apoptosis, reduce lipogenesis, and promote the browning of white adipose tissue to alleviate over-obesity but most recent studies have focused on the mitophagy and obesity-associated co-morbidities, and relatively few studies have investigated the specific mechanisms of the protective role in obesity.

### 4.2 Mitophagy and T2DM

T2DM is a common metabolic disease characterized by hyperglycemia, in which insulin resistance and impaired pancreatic β-cell function hinder its main drivers (Gouda et al., 2021). During the development of T2DM, pancreatic β-cells are often exposed to high concentrations of glucose, and the rapid accumulation of dysfunctional mitochondria accompanied by excessive ROS release can trigger oxidative damage or even death of pancreatic β-cells and exacerbate insulin resistance (Wang S. et al., 2023). Enhanced mitophagy significantly inhibits endoplasmic reticulum stress (ERS) and iron death and improves pancreatic β-cell function. Selenium Nanodots (SENDs) are precursor drugs for glutathione peroxidase 1 (GPX1), which can be obtained from dehydrated polymerization of selenocysteine under alkaline conditions. These are enriched in pancreatic islets and enter the β-cells to scavenge high levels of ROS. SENDs significantly upregulate the mRNA expression of GPX1 in pancreatic tissues of T2DM mice established using a high-fat diet and streptozotocin (STZ) treatment, increases protein expressions of Parkin, PINK1, and LC3-II/I, restores pancreatic mitophagy, and significantly reduces the mRNA expression of glucose-regulating protein 78 (GRP78), protein kinase R-like endoplasmic reticulum kinase (PERK), eukaryotic initiation factor-2α (eIF-2α), activating transcription factor 4 (ATF4) and C/BEBP homologous protein (CHOP), and effectively inhibits ERS and apoptosis. The β-cell function is restored in T2DM rats. In an *in vitro* model of pancreatic islet INS-1 cells in PA-treated rats, SENDs increase mRNA expression of GPX1, enhance the level of Parkin/PINK1-mediated mitophagy, downregulate GRP78, PERK, eIF-2α, and CHOP expression, and inhibited ERS and apoptosis, thereby maintaining the numerical and functional stability of pancreatic islet β cells (Huang et al., 2023). Silibinin is a flavonolignan analog extracted from the fruits and seeds of Silybum marianum. It can regulate insulin production and lipid synthesis (Cai et al., 2023). Silencing PINK1 significantly reduces Parkin expression in palmitic acid (PA) and high glucose (HG)-treated rat islet β-cell lines (INS-1), inhibits PINK1/Parkin-mediated mitophagy, and consequently downregulates inhibitory molecules of iron death, including glutathione (GSH), GPX4, and ferroptosis suppressor protein 1 (FSP1). This exacerbates iron death in INS-1 cells. Silibinin significantly upregulates PINK1 and Parkin expression in PA and HG-treated INS-1 cells, induces mitophagy, and downregulates the expression of total iron, lipid ROS, malondialdehyde (MDA), and COX-2, thus significantly increasing the expression of GSH, GPX4, and FSP1 and inhibiting PA- and HG-induced iron death in INS-1 cells, which in turn upregulate the expression of glucose transporter 4 (Glut4) and carnitine palmitoyltransferase 1 (CPT1), increase MMP and ATP production, inhibit ROS production, and improve glucose and FA metabolism in INS-1 cells (Du et al., 2023).

Activation of mitophagy alleviates insulin resistance and OS and improves T2DM. Compound K (CK) is a natural molecule with a steroid-like structure extracted from ginseng, which has been shown to possess several pharmacological activities for the treatment of T2DM (Tian et al., 2022). CK activates PINK1/Parkin-mediated mitophagy in the skeletal muscle of db/db mice, upregulates the expression of mitofusion 1 (MFN1), MFN2, and optical atrophy 1 (OPA1), decreases the expression of fission mitochondrial 1 (FIS1), inhibits the activity of Drp1, and upregulates the expression of FA oxidation protein acyl-co an oxidase 1 (ACOX1), carnitine palmitoyltransferase 2 (CPT2) and COX6A1, along with mitochondrial DNA content. This improves skeletal muscle mitochondrial mass reduces serum TG, TC, non-esterified fatty acid (NEFA), blood glucose, and insulin levels, and improves lipid metabolism, glucose tolerance, and insulin resistance in db/db mice. *In vitro*, CK activates PINK1/Parkin-mediated mitophagy in insulin-resistant cellular models established by FA and HG-treated mouse adult myoblasts (C2C12), promotes mitochondrial fission/fusion stabilization, and ameliorates FA-induced insulin resistance in skeletal muscle cells. The knockdown of Drp1 inhibits the activating effect of CK on skeletal muscle mitochondria, suggesting that CK activates Drp1/PINK1-mediated mitophagy, which in turn improves the mitochondrial quality and function and alleviates insulin resistance (Li W. et al., 2023). Cyanidin-3-O-glucoside (C3G) is a commonly studied anthocyanin with strong reducing power and antioxidant capacity (Wang B. et al., 2024). HG and PA treatment decrease mitochondrial MMP and increased ROS and O_2_ production in pancreatic islet cells (Net-1), resulting in the impairment of mitochondrial function. C3G increases the expression of PINK1 and Parkin in HG- and PA-treated Net-1 cells manifested as the accumulation of LC3 in Net-1 cells and activation of PINK1/Parkin-mediated mitophagy, which in turn inhibits the excess production of ROS and O_2_, while autophagy inhibitors exert an inhibitory effect on the antioxidant capacity of C3G. C3G intervention in animals significantly downregulates the level of ROS in the pancreas of diabetic db/db mice, upregulates the activities of superoxide dismutase (SOD) and catalase (CAT), alleviates the islet OS induced by hyperglycemia in diabetic mice, and ameliorates T2DM (Ye X. et al., 2021).

In summary, mitochondrial damage triggered by mitophagy dysfunction is closely related to T2DM, and activation of mitophagy can inhibit ERS, iron death, apoptosis, os, and insulin resistance, and reduce pathogenic risk factors of T2DM. However, most of the current studies focus on diabetic complications related research, revealing the pathophysiological mechanisms of mitophagy in T2DM are relatively few, and need to be further researched.

### 4.3 Mitophagy and metabolic-associated fatty liver diseases

Metabolic associated fatty liver disease (MAFLD) refers to a series of diseases caused by multi-systemic metabolic dysfunction resulting in chronic liver damage, including hepatic steatosis, NAFLD, hepatic fibrosis, liver cirrhosis, hepatocellular carcinoma, and others (Boeckmans et al., 2021). After MAFLD occurs, hepatic mitophagy is abnormal and produces excessive ROS, which leads to an imbalance of hepatic fat-energy metabolism and further promotes the development of hepatic inflammation and fibrosis (Clare et al., 2022). Studies have confirmed that improving impaired mitophagy reduces hepatic inflammation and OS and prevents pathological exacerbation of MAFLD. Corn peptide (CP) has a small molecular weight. CP constitutes highly active peptides extracted from corn protein powder, which is produced by enzymatic hydrolysis or microbial fermentation. It has gained widespread attention because of its multiple bioactive properties (Díaz-Gómez et al., 2017). CP can significantly upregulate the protein expression of PGC-1α, PPARα, PINK1, Parkin, ATG7, and LC3I/II in the liver of high-fat dilated-induced NAFLD rats, downregulate that of P62, activate mitophagy mediated by PINK1/Parkin, and block liver steatosis induced by high-fat diet in rats. *In vitro*, CP upregulates the protein levels of PGC-1α, PPARα, PINK1, Parkin, ATG7, and LC3I/II in free FA (FFA)-induced NAFLD cell models, activates the PINK1-Parkin signaling pathway to promote mitophagy in liver cells, and inhibits FFA-induced OS and lipid accumulation. Knocking down PINK1 effectively inhibits the hepatoprotective effects of CP (Yao Z. et al., 2023). Glial cell line-derived neurotrophic factor (GDNF) can significantly reduce MDA levels and inhibit OS in the liver of mice with liver injury induced by a Western diet, glucose, and fructose. In addition, GDNF significantly reduced ROS accumulation and mitophagy damage in the PA-induced hepatocyte injury model. *In vitro* experiments further confirmed that GDNF significantly increased SOD activity in the PA-induced hepatocyte injury model, and upregulated SIRT3, PINK1, and OPA1 protein levels, thereby promoting mitophagy. In turn, this enhanced the catalytic effect of SOD on ROS catabolism and inhibited hepatocyte OS (Mwangi et al., 2022).

Another study confirmed that enhanced mitophagy could reduce hepatic lipid accumulation and alleviate hepatic metabolic disorders. Zinc finger protein 143 (ZNF143) and lncRNA NEAT1 were upregulated in FFA-treated hepatocytes. ZNF143 knockdown significantly reduced lncRNA NEAT1 expression in hepatocyte injury models, inhibited the interaction between staphylococcal nuclease domain-containing protein 1 (SND1) and Rho-associated coiled coil-containing protein kinase 2 (Rock2), and decreased ROCK2 mRNA expression. Then, it upregulated the expression of PINK1, Parkin, Beclin-1, and LC3-II/I, downregulated sterol regulatory element binding transcription factor 1 (SREBP1), liver X receptors α (LXRα), and stearoyl-CoA desaturease1 (SCD1) expression, enhanced mitophagy, and attenuated hepatocyte steatosis. *In vivo* experiments demonstrated that knockdown of ZNF143 or lncRNA NEAT1 significantly upregulated hepatic PINK1, Parkin, Beclin1, and LC3II/I expression in high-fat diet-induced NAFLD mice, further validating the ameliorative role of the ZNF143/lncRNA NEAT1/ROCK2 signaling pathway in high-fat diet-induced hepatic tissue injury in mice (Dong et al., 2023). Quercetin (Que) is a natural herbal monocomponent that exerts hepatoprotective functions through anti-inflammatory effects (Turedi, 2023). Que significantly increased liver ATG5, ATG12, LC3, PINK1, and Parkin protein levels in NAFLD mice induced by methionine and choline deficiency (MCD) diet, promoted mitophagy, inhibited nucleotide-binding oligomerization domain-like receptor protein 3 (NLRP3) protein levels, reduced serum alanine aminotransferase (ALT)/aspartate aminotransferase (AST) and ROS levels, and alleviated hepatic atrophy and lipid accumulation in NAFLD mice. *In vitro* experiments further confirmed that Que significantly upregulated AMPK expression in oleic acid (OA)-stimulated hepatocytes, increased PINK1 and Parkin protein levels, and enhanced mitophagy, which subsequently inhibited NLRP3 and IL-1β expression, downregulated ROS production, and attenuated OA-induced hepatocyte injury (Cao et al., 2023).

In summary, enhanced mitophagy can reduce hepatic inflammation, OS, apoptosis, lipid accumulation, and steatosis, and inhibit the developmental process of MAFLD. However, there is a relative lack of literature related to the potential mechanisms of mitophagy and MAFLD, and future studies are still needed to explore their specific mechanisms in depth.

### 4.4 Mitophagy and CVDs

CVD is the leading threat to human health and causes death from non-infectious diseases worldwide (Virani et al., 2020). Mitochondrial dysfunction can cause uncoupling of the mitochondrial respiratory chains, which contributes to increased ROS production and decreased ATP synthesis. In turn, this leads to cardiomyocyte damage, induces apoptosis or necrosis, and exacerbates myocardial tissue damage (Li A. et al., 2023). Studies have shown that promoting mitophagy suppresses inflammatory responses and OS, which subsequently improves CVD. Ginsenoside is the main component responsible for the physiological activity of ginseng. Ginsenoside Rg3 is a tetracyclic triterpenoid saponin that exerts a wide range of pharmacological effects (Zhang C. et al., 2023). Ginsenoside Rg3 significantly increased the expression of ULK1 and FUNDC1 in heart failure (HF) rats’ hearts, promoted the interaction of FUNDC1 and LC3 to initiate mitophagy, reduced the number of inflammatory cell infiltration, infarct size, and serum biomarker expression in HF rats’ hearts, and significantly decreased the mRNA expression of natural peptide predictor a (NPPA), NPPB, and the protein levels of ColⅠ and ColⅡ. The levels of left ventricular end-diastolic dimension (LVIDD), left ventricular internal dimension system (LVIDS), left ventricular ejection fraction (LVEF), and left ventricular fractional shortening (LVFS) were also decreased, which could improve OS and mitochondrial damage in HF rats. *In vitro* experiments further confirmed that Rg3 treatment increased ULK1 and FUNDC1 expression in oxygen-glucose deprivation/reperfusion (OGD/R)-treated rat cardiomyocytes, triggered the phosphorylation of FUNDC1 at the Ser17 site via ULK1 to activate mitophagy, and facilitated FUNDC1-LC3 interactions, which resulted in the maintenance of mitochondrial homeostasis and energy metabolism and ameliorated HF (Wang X. et al., 2023). Xinmaikang (XMK), an herbal compound for the treatment of atherosclerosis (AS), contains four main herbs, namely, Campanulaceae (Dangshen), Rutaceae (Zhishi), Trionycis Carapax (Biejia), and Pheretima (Dilong). XMK treatment significantly downregulated serum levels of IL-6, IL-1β, monocyte chemoattractant protein-1 (MCP-1), and tumor necrosis factor-α (TNF-α) in high-fat diet feeding-induced AS mice. It also increased the expression of PINK-1, Parkin, lysosome-associated membrane protein 1 (LAMP1), and translocase of the outer mitochondrial membrane member 20 (TOMM20) in the aorta of AS mice, enhanced mitophagy in aorta-derived mitophagy, and reduced plaque, lipid, and ROS production in AS mice. *In vitro* experiments confirmed that XMK significantly upregulated the levels of PINK1, Parkin, TOMM20, and LC3 and decreased the level of SQSTM1 in oxidized low-density lipoprotein (ox-LDL)-treated macrophages. Activating PINK1/Parkin-mediated mitophagy in macrophages can prevent and treat AS (Cao Y. et al., 2023).

Other studies have shown that regulating mitophagy disorders can reduce cardiomyocyte damage and improve cardiac function. Overexpression of PINK1 can significantly upregulate Beclin1 and LC3 protein expression in an isoproterenol (ISO)-induced mouse cardiomyocyte injury model, decrease p62 protein expression, enhance myocardial mitophagy, and alleviate ISO-induced MMP reduction, ROS production, and cell apoptosis. Overexpression of Mfn2 can enhance mitochondrial fusion and improve ISO-induced mitochondrial fission. Combined treatment of metformin and PINK1/Mfn2 overexpression significantly reduced ROS production and apoptosis, inhibited β-myosin heavy chain (β-MHC) expression, increased mitochondrial MMP, and improved myocardial injury. *In vivo* experiments showed that overexpression of PINK1 reduced the mean cross-sectional area of cardiomyocytes in mice with transverse aortic constriction (TAC)-induced myocardial injury, downregulated the levels of atrial natriuretic peptide (ANP), β-MHC, Col Ⅰ, transforming growth factor-β1 (TGF-β1), and p62 protein levels, upregulated Beclin1 and LC3 protein expression, promoted myocardial mitophagy, and ameliorated cardiac hypertrophy and fibrosis in TAC mice (Ma Z. et al., 2023). Hypoxia/reoxygenation (H/R)-induced cardiomyocyte injury model showed significant downregulation of miR-494-3p mRNA expression, upregulation of PGC-1α, PINK1, and Parkin protein expression, downregulation of Mfn1, Mfn2, and OPA1 protein expression, and reduction of mitochondrial MMP, suggesting that H/R induced excessive mitophagy in cardiomyocytes. Further experiments demonstrated that overexpression of miR-494-3p could target and negatively regulate the expression of PGC-1α. Moreover, it inhibited the expression of LC3, Beclin1, PINK1, and Parkin, and promoted the expression of Mfn1, Mfn2, and OPA1. Downregulating the expression of Bax and upregulating the expression of Bcl-2 can inhibit excessive myocardial mitophagy, reduce cell apoptosis, and thus improve myocardial cell injury (Mu et al., 2023).

In summary, ginsenoside Rg3, XMK, PINK1, and miR-494-3p can inhibit cardiomyocyte apoptosis, inflammatory response, OS, and fibrosis by regulating mitophagy and delaying the development of CVD. However, the study of mitophagy and CVD is only limited to cardiomyocytes, and it may play a protective role in other heart cells, such as myocardial fibroblasts and endothelial cells (ECs), but the mechanism is still unclear.

### 4.5 Mitophagy and diabetes complications

Diabetes mellitus is a type of metabolic disease caused by abnormal lipid metabolism or insulin dysfunction, and persistent chronic elevation of blood glucose and long-term metabolic disorders can damage tissues and organs throughout the body, leading to complications such as diabetic kidney disease (DKD), diabetic cardiomyopathy (DCM), and diabetic muscle atrophy (Wen et al., 2023). Under HG conditions, the mitochondrial respiratory chain releases excess ROS to the cytoplasm to signal hypoxia, triggering mitochondrial depolarization damage. In turn, this inhibits respiratory chain activity and mitophagy and induces oxidative damage and apoptosis in pancreatic islet β-cells, leading to enhanced insulin resistance (Čater and Križančić, 2022). Studies have confirmed that activating mitophagy can inhibit apoptosis and inflammatory responses and delay the occurrence and development of diabetes complications. Poricoic acid A (PAA) is a tetracyclic triterpenoid extracted from Poria cocos, It possesses biological activities such as antioxidant, anti-inflammatory, and hypoglycemic properties (He Y. et al., 2022). HG treatment upregulates the protein expression of FUNDC1 in mouse podocytes, promotes FUNDC1-mediated mitophagy, enhances the expression of IL-6 and IL-1β, decreases podocyte viability, proliferation, and mitochondrial MMP levels, increases ROS generation, and accelerates podocyte injury in mice. PAA significantly increased the levels of LC3 and ATG5, decreased the levels of p62 and FUNDC1 proteins, induced mitophagy, decreased ROS production, increased MMP levels, inhibited the expression of IL-1β, IL-6, and TNF-α, and significantly inhibited the apoptosis and inflammatory response of HG-induced podocytes in mice. *In vivo* experiments further confirmed that PAA significantly reduced blood urea nitrogen (BUN) and proteinuria levels in STZ-induced DKD mice, inhibited the expression of IL-1β and TNF-α in renal tissues, significantly increased the levels of LC3 and ATG5 proteins, and downregulated the levels of marker proteins such as p62 and FUNDC1, suggesting that PAA can improve renal dysfunction in DKD mice by downregulating FUNDC1 activation of mitophagy (Wu et al., 2023). Metformin is a commonly used drug for the clinical treatment of T2DM, which improves the sensitivity and utilization of insulin in body tissues and reduces the occurrence of adverse events such as hypoglycemia and obesity (Pernicova and Korbonits, 2014). Metformin significantly upregulated the levels of p-PINK1, PINK1, Parkin, and p62 and increased the ratio of LC3-Ⅱ/LC3-Ⅰ in the brain tissue of diabetes rats with CI/R injury. Moreover, it decreased the level of Drp1, increased the expression of Bcl-2, and decreased the expression of Bax and cytochrome c (Cyt-c). Additionally, metformin decreased the ratio of Cleared Caspase-3/Caspase-3, Cleaved Caspase-9, activated PINK1/Parkin mediated mitophagy, and prevented mitochondrial over fission and apoptosis. Cell experiments further confirmed that metformin treatment significantly enhanced the expression and phosphorylation of AMPK and ULK1 in mouse hippocampal neuronal cell injury models treated with HG and OGD/R, thereby activating PINK1/Parkin mediated mitophagy. This suggests that metformin may alleviate CI/R damage by activating the AMPK/ULK1/PINK1/Parkin mitophagy pathway (Guo et al., 2023).

Studies have demonstrated that enhanced mitophagy can improve EC dysfunction and OS and alleviate the complications of diabetes. L-carnitine, a water-soluble quaternary ammonium compound, is an essential factor for the entry of long-chain FAs into the mitochondrial matrix (Ferreira and Kenna, 2017). L-carnitine could significantly reduce the levels of TG, ceramide, and FFA in the heart of DCM mice, increase the expression of CPT1A and GLUT4, reduce the expression of intercellular adhesion molecule-1 (ICAM-1) and vascular cell adhesion molecule-1 (VCAM-1), inhibit lipid peroxidation, enhance endothelial barrier function, and maintain the microvascular structure of DCM mice. *In vitro* experiments showed that PARL segregated from PHB2 in an *in vitro* model of HG/FFA-induced cardiac microvascular ECs in mice, decreased the expression of PINK1, Parkin, and LC3BII, reduced the co-localization of Parkin and mitochondria, inhibited mitophagy, and accelerated the injury of cardiac microvascular ECs in mice. L-carnitine treatment significantly enhanced the expression of CPT1A in Hg and FFA-treated mouse cardiac microvascular ECs, promoted the interaction between its downstream PHB2 and PARL, and then enhanced PINK1/Parkin-dependent mitophagy, inhibited the release of Cyt-C and the activation of Caspase-3, and improved mitochondrial dysfunction and cardiac microvascular damage in DCM (Li S. et al., 2023). Psoralea corylifolia L. seed (PCS) is widely used in Korean traditional medicine, and two compounds isolated from PCS, namely, bavachin and corylifol A, have myogenic activity and are the main components of traditional herbal prescriptions for musculoskeletal disorders (Han Y. et al., 2020). Bavachin and Corylifol A administration significantly inhibited NF-κB phosphorylation and its downstream TNF-α and IL-6 protein levels in muscle tissues of diabetic muscular dystrophy mice. Additionally, it downregulated the expression of myostatin (MSTN), atrogin-1, and muscle-specific RING finger protein 1 (MuRF-1), activated the Akt synthesis signaling pathway, reduced blood glucose level, and increased the muscle mass of diabetic muscular dystrophy mice. In addition, Bavachin and Corylifol A administration can activate mitophagy, inhibit OS, and thus improve mitochondrial quality by upregulating LC3-II, p62, Parkin, PINK1, and BNIP3 protein expression in muscle tissues of diabetic muscular dystrophy mice (Yeon et al., 2023).

In summary, mitophagy mediated by PINK1/Parkin and FUNDC1 is an important defense mechanism for diabetic complications, which can alleviate the damage associated with diabetic complications by inhibiting OS, the inflammatory response, and apoptosis, and improving the barrier function of ECs. However, most of the current studies have focused on the PINK1/Parkin-dependent mitophagy pathway, and more research needs to be conducted on other receptor-dependent pathways, which need further exploration.

### 4.6 Mitophagy and NDDs

NDD is a class of metabolic diseases in which the gradual loss of neurons and their myelin sheaths leads to central and peripheral nervous system dysfunction, seriously affecting patients’ health and quality of life (Lu et al., 2022). Mitophagy usually induces an abnormal state during the occurrence and development of NDD, leading to excessive accumulation of dysfunctional mitochondria in neurons, triggering neuronal necrosis and OS, and aggravating neuronal damage (Goldsmith and Holzbaur, 2022). Research has shown that improving mitophagy abnormalities can alleviate nerve damage caused by the accumulation of misfolded proteins and delay the pathological process of NDD. MORIN, a flavonoid found in mulberry plants or other herbs, has a variety of effects, including anti-inflammatory, antioxidant, and neuroprotective properties (Elizabeth et al., 2020). MORIN dephosphorylated and activated transcription factor EB (TFEB) in a mouse brain tumor neuronal cell line (N2a cell line), which subsequently translocated TFEB to the nucleus and increased autophagy marker proteins PINK1, Parkin, LC3-II, AMPK, and ULK2 expression in a time-dependent and dose-dependent manner. Downregulation of Mfn1 and TOMM20 expression activates the AMPK/ULK2 signaling pathway and induces mitophagy. *In vivo* experiments demonstrated that MORIN treatment significantly upregulated the expression of Parkin and LC3-II in the frontal cortex and hippocampus of Parkinson’s disease (PD) mice, downregulated the expression of Mfn1, increased the expression of tyrosine hydroxylase (TH) protein, facilitated mitophagy, attenuated the loss of dopaminergic neurons, and ameliorated the behavioral deficits in PD mice (Wang ZY. et al., 2023). Loganin is the main active ingredient extracted from Cornus officinalis, which ameliorates nerve damage (Cheng et al., 2021). Loganin significantly upregulated postsynaptic density-95 (PSD95), synaptophysin (SYP), Mfn2, and OPA1 protein levels in the hippocampus of 3×Tg-alzheimer disease (AD) mice, downregulated Drp1, Fis1, LC3II, p62, PINK1, Parkin, TOMM20, and COX isoform IV expression, improved amyloid β-protein (Aβ) deposition and synaptic ultrastructure in 3×Tg-AD mice, restored mitochondrial fission/fusion homeostasis, alleviated the abnormal degradation of mitophagy, and restored the learning and memory ability of 3×Tg-AD mice. *In vitro* experiments further confirmed that Loganin significantly upregulated OPTN expression and downregulated LC3II, p62, PINK1, and Parkin protein expression in Aβ_25-35_-treated human neuroblastoma cells (SK-N-SH), and significantly ameliorated mitochondrial dysfunction, whereas OPTN silencing counteracted the restoration of mitochondrial function by Loganin, confirming that Loganin may improve cognitive function in AD mice by promoting OPTN-mediated mitophagy (Zhou Y. et al., 2023). Melatonin, an endogenous circadian indoleamine secreted by the pineal gland, has a wide range of biological functions, including antioxidant, anti-inflammatory, antitumor, and neuroprotective effects (Porfirio et al., 2017). Ropivacaine treatment significantly upregulates the LC3-II/LC3-I ratio and the levels of Beclin1, PINK1, and Parkin proteins in rat PC12 cells and hippocampal neuronal cells (HT22), decreases the expression of p62, TOM20, and COXIV proteins, promotes mitochondrial hyperautophagy, increases the expression of pro-apoptotic proteins Bax and cleaved-caspase-3, decreases the expression of anti-apoptotic protein Bcl-2, reduces mitochondrial MMP, increases ROS, and induces apoptosis in PC12 and HT22 cells. Melatonin pre-treatment downregulates the expression of PINK1 and Parkin proteins, upregulates the levels of Tom20 and COXIV proteins, and inhibits Ropivacaine-induced mitochondrial hyperautophagy, which in turn reduces the Bax/Bcl-2 ratio and caspase-3 expression, and inhibits apoptosis in PC12 and HT22 cells (Zeng et al., 2022).

Other studies have confirmed that enhanced mitophagy can alleviate the deposition of aβ extracellular amyloid plaques and the loss of dopaminergic neurons, thus alleviating nerve damage. Polygala saponins (PSS), the main characteristic components of the advanced Chinese medicine Radix Polygalae, accelerate the autophagic degradation of specific proteins (Wu A. et al., 2013). PSS significantly promoted src homology phosphotyrosyl phosphatase 2 (SHP-2) and AMPK phosphorylation in BV-2 cells and decreased mammalian target of rapamycin (mTOR) and p70 ribosomal S6 kinase (P70S6K) phosphorylation, thereby upregulating PINK1 and Parkin protein expression, promoting LC3-I to LC3-II conversion, increasing LC3 co-localization with mitochondria, and activating mitophagy mediated by AMPK/mTOR and PINK1/Parkin signaling pathways. PSS upregulated SHP-2 expression in Aβ_1-42_-treated BV-2 cells to activate mitophagy, which subsequently downregulated NLRP3, Apoptosis-associated speck-like protein (ASC), IL-1β, IL-18, and Gasdermin D (GSDMD) expression, and inhibited NLRP3 inflammasome activation. Moreover, PSS significantly increased Aβ and Tau, p-SHP-2, and LC3II protein expression in brain tissue of AD mice and activated mitophagy, which subsequently inhibited NLRP3 inflammasome activation and improved cognitive function in AD mice (Qiu et al., 2022). CircEPS15 was significantly downregulated in plasma samples from PD patients and 1-methyl-4-phenyl-1,2,3,6-tetrahydropyritine (MPTP)-induced PD mice, and circEPS15 levels were correlated with H-Y staging/PD composite rating scale scores. Furthermore, overexpressed circEPS15 could act as a miR-24-3p sponge, inhibit miR-24-3p expression in 1-methyl-4-phenyl pyridine (MPP^+^)-treated human neuroblastoma cells (SH-SY5Y cells), upregulate the expression of the target gene PINK1, decrease the protein level of SQSTM1/p62, upregulate LC3 expression, and enhance PINK1-Parkin-dependent mitophagy, which subsequently upregulated Bcl-2 and TOMM20 expression, and reduced BAX and Cleaved-Caspase-9 expression, preventing MPP^+^-induced neuronal damage. *In vivo* experiments further confirmed that overexpression of circEPS15 significantly inhibited MPTP-induced PD in mice brain tissues with miR-24-3p expression, upregulated PINK1 expression, and enhanced PINK1-Parkin-dependent mitophagy to promote dopaminergic neuronal recovery in PD mice (Zhou YZ. et al., 2023).

In summary, direct or indirect regulation of mitophagy abnormality can alleviate the neurological damage caused by aβ extracellular amyloid plaque deposition, α-synaptic nucleoprotein accumulation, dopaminergic neuron loss, neuroinflammation, and OS, and play an essential role in the physiopathological process of NDD. However, there are more studies on mitophagy related to AD and PD and few on other NDD-related mechanisms.

## 5 Mitophagy and exercise intervention

**FIGURE 3:**
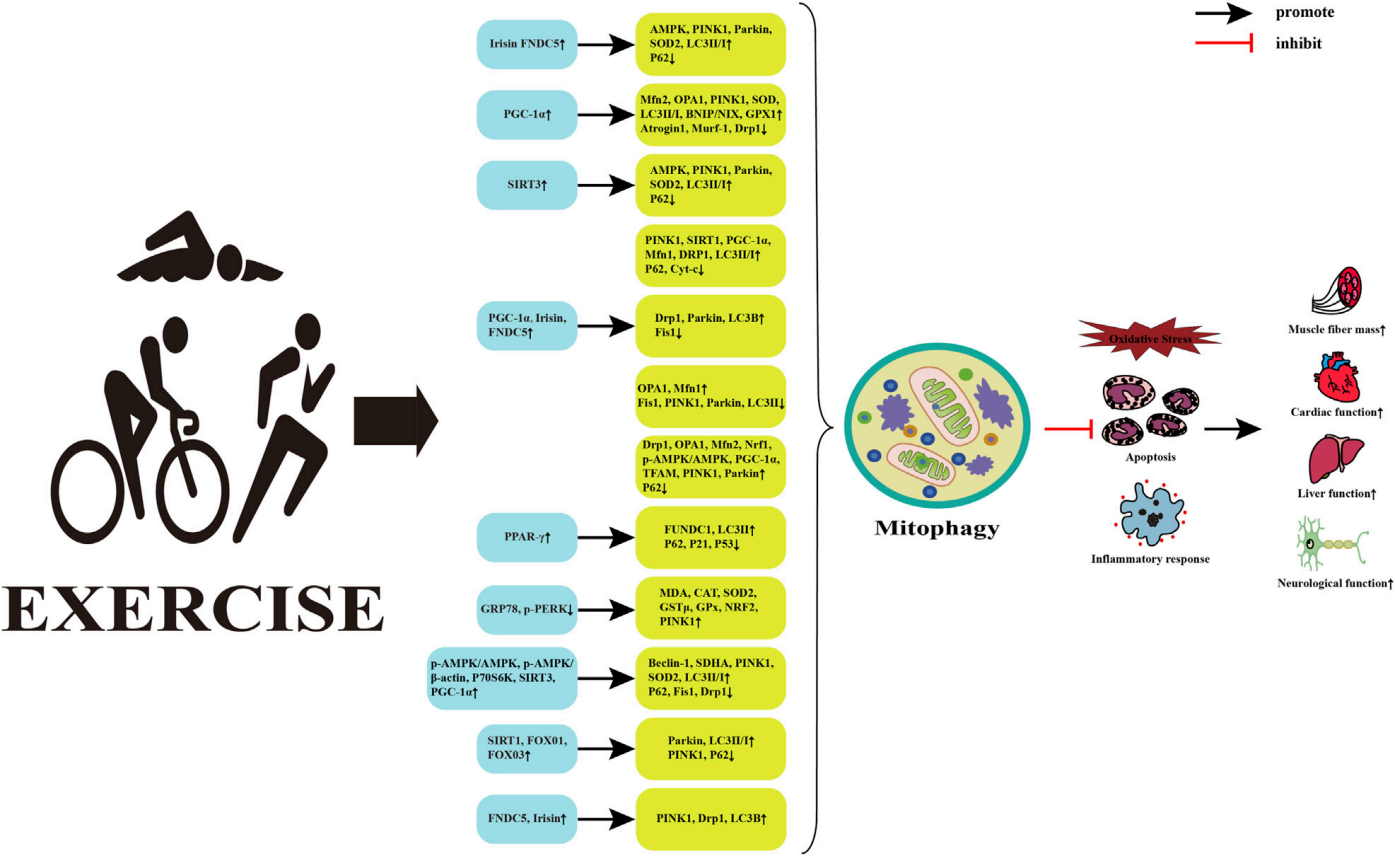
Potential mechanisms of exercise regulated mitophagy intervention in metabolic diseases. Abbreviations: FNDC5, fibronectin typeⅢdomain containing 5; AMPK, AMP-activated protein kinase; PINK1, PTEN induced putative kinase 1; SOD2, superoxide dismutase 2; LC3, microtubule-associated protein 1 light chain 3; PGC-1α, peroxisome proliferator-activated receptor γ coactivator 1α; Mfn2, mitofusin 2; OPA1, optic atrophy 1; BNIP, Bcl-2 adenovirus/E1B 19k D interacting protein; NIX, nip3-like protein; GPX1, glutathione peroxidase 1; Murf1, muscle-specific RING finger protein 1; Drp1, dynamin-related protein 1; SIRT3, silent information regulator 3; Cyt-c, Cytochrome c; Fis1, fission mitochondrial 1; Nrf1, nuclear respiratory factor 1; TFAM, mitochondrial transcription factor A; PPAR-γ, Peroxisome proliferator-activated receptors; FUNDC1, FUN14 do-main-containing protein 1; GRP78, Glucose-regulating protein 78; p-PERK, phosphorylation protein kinase-like ER kinase; MDA, malondialdehyd; CAT, catalase; GSTμ, glutathione S-transferase class μ; P70S6K, p70 ribosomal s6 kinase; Beclin-1, B-cell lymphoma-2-interacting myosin-like coiled-coil protein 1; SDHA, succinate dehydrogenase complex subunit A; FOXO1, forkhead transcription factor; ↑, upregulated; ↓, downregulated.

Mitophagy is one of the fundamental mechanisms for maintaining mitochondrial quality and cellular homeostasis. It can selectively remove severely damaged mitochondria and play a beneficial role in metabolic diseases. Physical activity is considered the cornerstone for preventing and treating metabolic diseases and can intervene in the physiological and pathological processes of metabolic diseases by regulating mitophagy (Memme et al., 2021) (Figure 3). However, the effects of different exercise types on mitophagy and the potential mechanism by which exercise regulates mitophagy to intervene in metabolic diseases remains unclear.

### 5.1 Effects of different exercise types on mitophagy

#### 5.1.1 Aerobic endurance exercise intervention

Aerobic endurance exercise upregulates mitophagy. Four weeks of autonomous running wheel exercise significantly upregulates protein levels of LC3BII, Parkin, p62, PGC-1α, and mitochondrial fission factor 1 (MFF1) phosphorylation in the mitochondria of the hearts of A-kinase-interacting protein 1 (AKIP1) overexpressing mice. It downregulates the protein expression of NRF2, with no significant changes in the levels of OPA1 and Mfn1 proteins. It promotes mitophagy and fission in the mouse heart, inhibits mitochondrial biogenesis, and thus, significantly increases the number of cardiac mitochondria, reduces the average mitochondrial volume, and promotes post-exercise mitochondrial turnover (Nijholt et al., 2023). Sixteen weeks of running wheel exercise upregulate the expression of adiponectinreceptor1 (AdipoR1) protein in the skeletal muscle of aged mice, promotes its downstream protein’s transcriptional activity, forkhead box class O3 (FOXO3) to increase FOXO3a protein levels, which in turn upregulates LC3-II and BNIP3 protein expression, downregulates p62/SQSTM1 protein levels, and increases the mRNA expressions of Beclin1, ULK1, recombinant phosphoinositide-3-kinase class 3 (Pik3c3), ATG5, ATG7, LC3B, BNIP3, and PINK1. This induces autophagy and mitophagy in the skeletal muscle of aged mice and improves muscle mass. Eight days of swimming exercise significantly increases the expression of the decay-accelerating factor 16 (DAF-16) in the L4-stage hidradenitis elegans nematodes, followed by PINK1 mRNA expression, which prolongs the lifespan of hidradenitis elegans nematodes by promoting autophagy and mitophagy (Chen Y. et al., 2023). One-time force exhaustion exercise significantly increases protein expressions of BNIP3, PARK2, PINK1, Drp1, and Mfn2 and Drp1 phosphorylation. It increases TFEB myonuclear localization in skeletal muscle of myotonic dystrophy type 1 (DM1) mice, downregulates LC3-I, LC3-II, and p62 protein levels, promotes autophagy and mitophagy in the skeletal muscle, enhances skeletal muscle mitochondrial biogenesis, coordinates mitochondrial fusion and fission, and improves the overall health of skeletal muscle in DM1 mice (Mikhail et al., 2023). Six weeks of hypoxic treadmill exercise significantly increases Sirt3 expression in the skeletal muscle of C57BL/6J mice, followed by upregulating the levels of FOXO3a, SOD2, PINK1, and Parkin proteins, with no significant change in the expression of Drp1 proteins, which promotes the level of mitophagy, and consequently improves the synthesis of ATP and the activity of citrate synthase (CS) in the skeletal muscles and increases mitochondrial volume and number. After intraperitoneal injection of the Sirt3 inhibitor in C57BL/6J mice, levels of FOXO3a protein decrease and mitochondria show pathologic damage, indicating that inhibition of Sirt3 expression significantly impaired mitochondrial function (Ma C. et al., 2022). In contrast to these studies, aerobic endurance exercise negatively modulates mitophagy levels. Eight weeks of aerobic endurance exercise and choline injection intervention increase myocardial M_2_Acetylcholine receptor (M_2_AChR) expression in TAC surgery-induced cardiac hypertrophy mice, followed by downregulation of Mfn1, Mfn2, Drp1, LC3, BNIP3, and Parkin proteins, with no significant changes in the levels of the OPA1 protein. It reduces excessive mitophagy and fission in hypertrophied mice, resulting in an improvement in the ultrastructure and function of myocardial mitochondria after TAC. Eight-week aerobic endurance exercise and choline injection interventions upregulate myocardial M2AChR expression and inhibit MFN2, PERK, eIF-2α, ATF4, NLRP3, caspase-1, and IL-1β proteins in cardiomyopathic hypertrophied mice, which effectively attenuate myocardial ERS and inflammatory responses, thereby improving cardiac function (Ma M. et al., 2021).

#### 5.1.2 High-intensity interval exercise intervention

High-intensity interval training promotes mitophagy levels. Four weeks of high-intensity interval exercise increases the levels of PGC-1α, mitochondrial respiratory complex II/III proteins, inhibits hyperphosphorylation of Src Tyr416 and Akt Ser473 and the level of p62 protein in the skeletal muscle of dystrophin-deficient mdx52 mice. It upregulates the expression of Pink1, Parkin, Bnip3 and BCL2 like 13 (Bcl2l13) proteins and LC3B-II/I ratio, promotes mitophagy in the skeletal muscle of mdx52 mice, and significantly inhibits the expression of inflammatory and OS marker proteins (CD68, CD206, SOD1, and SOD2), which in turn improve the anti-fatigue ability of skeletal muscle of mdx52 mice. A single acute high-intensity interval exercise increases AMPK, Acetyl CoA carboxylase (ACC), Ulk1 protein expression, and Drp1 phosphorylation, promotes mitochondrial fission and mitophagy, and improves the anti-fatigue ability of skeletal muscle of mdx52 mice (Yamauchi et al., 2023). A 12-week high-intensity interval exercise intervention enhances TFAM, TOM20, MFN2, and Parkin protein levels in the skeletal muscle of obese older adults without significant changes in Drp1 protein expression, increases skeletal muscle mitochondrial content and mass control, and improves physical function, muscle strength, body composition, and blood parameters (Youssef et al., 2022). L-Citrulline (CIT) is a non-proteinogenic amino acid, a by-product of nitric oxide production from L-arginine (Papadia et al., 2018). A 12-week high-intensity interval training intervention (oral and non-oral CIT) upregulates TFAM, TOM20, OPA1, Mfn1, Mfn2, and Parkin protein expressions in the skeletal muscle of obese older adults but Drp1 protein levels remain unaffected. It promotes the levels of mitochondrial biogenesis, mitochondrial fusion, and mitophagy in skeletal muscle, increases mitochondrial number and content in the skeletal muscle, and effectively improves lower limb muscle strength, skeletal muscle mass, body weight, and physical function (Marcangeli et al., 2022). High-intensity interval training reduces mitophagy. Seven weeks of high-intensity interval training downregulates hippocampal cAMP response element binding protein (CREB) phosphorylation and brain-derived neurotrophic factor (BDNF) protein levels, upregulates the protein expression of mitochondrial respiration Complex III, Complex IV, and Complex V, reduces those of Complex I, LC3-II and Bnip3L and LC3-II/Ⅰ ratio, and inhibits hippocampal mitophagy, which in turn decreased spontaneous behavior and exploratory activities and increases anxiety levels in middle-aged mice (Zhang Y. et al., 2021).

#### 5.1.3 Resistance exercise intervention

Resistance training positively modulates mitophagy. After two consecutive weeks of unilateral lower extremity immobilization, walking recovery, and resistance exercise interventions in healthy middle-aged men, a significant downregulation of PGC-1α, mitochondrial fission process 1 (MTFP1), TFAM, and DRP1 mRNA levels was found, along with a slight decrease in NRF1 and MFN1 protein levels in the skeletal muscle tissue of middle-aged men during the period of unilateral lower extremity immobilization. Resistance exercise significantly upregulates mRNA expressions of OPA-1, MFN2, FIS1, DRP1, MTFP1, and PINK1 and increases those of NRF1, OPA-1, FIS1, and Parkin proteins in skeletal muscle tissues, suggesting that resistance exercise promotes mitochondrial biogenesis, fission, fusion, and mitophagy and restores the levels of the marker proteins to their pre-intervention levels (Pileggi et al., 2023). A 9-week resistance training intervention reduces the accumulation of Aβ in skeletal muscle of inclusion body myositis (IBM) rats induced by peritoneal injection of chloroquine, decreased the expression of skeletal muscle mitochondrial Bax, Cyt-C, and cleaved caspase-3 proteins, and upregulates the expression of Bcl-2 proteins. It inhibits apoptosis of skeletal muscle mitochondria in IBM rats, upregulates the levels of PGC-1α, NRF-1, NRF-2, Tfam, TOM40, TOM20 and TIM-related protein 23 (TIM-23), promotes mitochondrial biogenesis in IBM rat skeletal muscle, upregulates the expression of PINK1 and Parkin proteins, downregulates the LC3-II/LC3-I ratio and the levels of p62 proteins, increases the levels of IBM rat skeletal muscle mitophagy level, decreases IBM rat skeletal muscle Drp-1 and Fis-1 protein expression, upregulates the levels of SIRT3 and its downstream protein FOXO3a, and simultaneously increases SOD-2 and CAT expression, along with improving the mitochondrial dynamics and antioxidant capacity of IBM rat skeletal muscle (Koo et al., 2019). Spermidine (SPD) is a polyamine that acts as a natural inducer of autophagy (Zhang Y et al., 2023). Six-week intraperitoneal injection of SPD and resistance training increase the p-AMPKα/AMPKα ratio and FOXO3a protein expression in skeletal muscle of D-galactose (D-gal)-induce senescent muscular dystrophy rats, activate the AMPK-FOXO3a signaling pathway, upregulate the levels of Beclin1 protein and the LC3-II/LC3-I ratio, and downregulate those of the p62 protein. It increases the Bcl-2/Bax ratio and decreases Bax and cleaved-caspase-3 protein levels, which in turn promotes skeletal muscle autophagy and mitophagy, inhibits apoptosis, and improves the functional status of skeletal muscle mitochondria and skeletal muscle atrophy (Fan et al., 2017). In contrast to the results of these studies, mitophagy levels do not change after short-term resistance training. After the short-term resistance training intervention, no significant changes were observed in the expression of MQC-associated markers in the skeletal muscle of older adults, including mitochondrial fission and fusion (Drp1, OPA1, and MFN2), mitochondrial biogenesis (PGC-1α, TFAM, and ULK1), and mitophagy (BNIP3L/Nix and LC3B I/II) (Marshall et al., 2022).

#### 5.1.4 Multi-exercise type intervention

Exercise interventions with two or more types upregulate mitophagy levels. One-time exhaustion exercise (forced weight-bearing swimming until exhaustion) reduces LC3-I to LC3-II conversion, downregulates PNIK1, SIRT1, PGC-1α, Mfn1, and Drp1 protein expression, and upregulate p62 protein expression in the hearts of obese mice induced by high-fat diet. These result in impaired cardiac mitophagy, deficient mitochondrial biogenesis, and impaired mitochondrial fission/fusion. Eight-week swimming exercise pre-conditioning attenuates one-time exhaustion exercise-induced MQC deficits in the heart of obese mice, increases PINK1, SIRT1, PGC-1α, Mfn1, and Drp1 protein expression, decreases p62 and Cyt-c protein levels, promotes mitophagy, mitochondrial biogenesis, and mitochondrial fission/fusion homeostasis, which in turn downregulates Bax protein expression, increases Bcl-2 and Bcl-2/BAX levels, and inhibits cardiac apoptosis in obese mice (Dun et al., 2023). After combined intervention with low-intensity continuous swimming exercise, high-intensity continuous swimming exercise, and high-intensity interval swimming exercise in male Swiss mice, the expression of PGC-1α mRNA in the gastrocnemius muscle of the exercise group increases at 6, 12, and 24 h post-exercise compared with that in the sedentary group. Mfn2 mRNA expression reaches its peak at the immediate post-exercise time in the two high-intensity exercise groups. Drp1 mRNA expression increases 6 h post-exercise in the two continuous swimming exercise groups, and Drp1 mRNA is suppressed immediately and 12 h post-exercise in the high-intensity intermittent swimming exercise. High-intensity continuous swimming exercise uniquely induces the upregulation of PARK2 mRNA expression and increases the level of mitophagy, suggesting that different types of exercise and the duration of the measurements differently affect mitochondrial function and self-repair capacity (Pires et al., 2022). Young and aged female rats were subjected to 8 months of moderate-intensity continuous aerobic training and high-intensity interval training interventions. High-intensity interval training significantly increased p-AMPK/β-actin, p-AMPK/AMPK, and p-AMPK/COX-IV ratios, and upregulated Beclin-1, LC3-II, Parkin, PINK1, PGC-1α, and p70S6K protein levels and LC3-II/LC3-I ratio. It increased the upregulation of SOD2 mRNA and mitochondrial respiratory chain supercomplex (mitoSC) levels, and mRNA levels of IL-15, and OPA1, suggesting that high-intensity interval training can improve mitochondrial function in rat skeletal muscle mitochondria by activating the AMPK pathway to increase the level of mitochondrial biogenesis and mitophagy, promote the assembly and formation of mitochondrial stem cells, and inhibit OS (Han C. et al., 2022). After 8 weeks of moderate-intensity continuous training and high-intensity interval training in myocardial infarction (MI) mice and TAC mice established by left anterior descending ligation, cardiac microtubule-associated protein 1 light chain 3 beta (MAP1LC3B) mRNA expression and LC3II/Glyceraldehyde 3-phosphate dehydrogenase (GAPDH) and LC3II/LC3I ratios increased in the hearts of myocardial infarcted mice and TAC mice. The levels of PINK1, Parkin, Becn1, and hypoxia-inducible factor 1α (HIF-1α) mRNA increased. High-intensity interval training increased autophagy and mitophagy flux in MI mice, while moderate-intensity continuous training more significantly increased autophagy and mitophagy levels in TAC mice (Guo C. et al., 2022).

### 5.2 Potential mechanisms of exercise-regulated mitophagy intervention in metabolic diseases

#### 5.2.1 Exercise regulates mitophagy to inhibit OS

OS is a process in which the production of ROS in an organism exceeds the scavenging capacity of the antioxidant system, resulting in damage to cells and tissues (Stavely et al., 2023). It was demonstrated that 4 weeks of resistance exercise intervention significantly upregulated the mRNA and protein expression of Irisin and Fibronectin type III domain containing 5 (FNDC5) in the hearts of mice with MI, increased AMPK phosphorylation and SOD2 expression, promoted PINK1 and Parkin protein expression, and decreased p62 protein level, which subsequently promoted PINK1/Parkin-mediated mitophagy, inhibited OS, decreased myocardial LVIDs and LVIDD, increased the ejection fraction (EF) and the shortening fraction of the left ventricle (FS), and improved the cardiac function of mice. Cellular experiments further confirmed that the L-OPA1/S-OPA1 ratio and Irisin/FNDC5 expression were significantly upregulated in 5-aminoimidazole-4-carboxamide ribonucleotide (AICAR)-treated cardiomyocytes, which significantly increased PINK1/Parkin, SOD2 expression, and LC3II/I ratio, decreased p62 expression, and increased mitophagy and antioxidant capacity in AICAR-treated cardiomyocytes (Li H. et al., 2021). Extra virgin olive oil (EVOO) is the main edible fat of the Mediterranean diet, which is rich in various nutrients and has potent antioxidant and anti-inflammatory properties (Bendini et al., 2007). EVOO combined with endurance exercise training significantly increased PGC-1α, COX IV, and Cyt-c expression in gastrocnemius muscle of rats fed a high-fat diet, upregulated Mfn2, OPA-1, PINK1, Beclin-1, LC3II/I, BNIP/NIX, SOD, and GPX1 protein levels, and downregulated Atrogin-1, MuRF-1, and Drp1 expression and FoxO3 phosphorylation/dephosphorylation ratio, which subsequently promoted mitochondrial biogenesis, mitophagy, and mitochondrial fusion/fission homeostasis, significantly inhibited rat gastrocnemius muscle protein ubiquitination and OS, and ameliorated mitochondrial dysfunction in rats (Yeo et al., 2022). Eight weeks of short-term aerobic exercise significantly upregulated SIRT3 expression, decreased Mfn1, Mfn2, Drp1, and LC3-II protein expression, and increased Opa1, PINK1, and Parkin protein levels in the hearts of aging MI mice. Moreover, it promoted mitophagy and mitochondrial fission/fusion homeostasis, increased SOD2 expression, downregulated ROS production, inhibited myocardial cell apoptosis and OS, and prevented MI injury in aging mice. Cellular experiments further confirmed that silencing SIRT3 expression significantly promoted hypoxia- and doxorubicin (DOX)-induced cardiomyocyte apoptosis and lactate dehydrogenase (LDH) release, upregulated SOD2 expression and increased ROS release, inhibited Opa1, PINK1, and Parkin protein expression, and significantly increased Drp1, LC3- II, and p62 expression, suggesting that short-term aerobic exercise mediates SIRT3 protection against aging myocardial injury (Zhao D. et al., 2018). In summary, different forms of exercise can regulate mitophagy to inhibit OS and improve the pathological state of damaged tissue cells.

#### 5.2.2 Exercise regulates mitophagy to inhibit apoptosis

Apoptosis is one of the essential forms of cell death, and inhibiting apoptosis can slow the occurrence and development of metabolic diseases (Kaur et al., 2023; Pei et al., 2023). A study confirmed that 8 weeks of swimming exercise significantly reduced serum fasting blood glucose (FBG), insulin, and homeostatic model assessment of insulin resistance (HOMA-IR) levels in high-fat diet-induced obese mice, lowered body weight, visceral fat content, and visceral fat content/body weight ratio, and inhibited obesity development. In addition, swimming significantly increased the LC3-I to LC3-II conversion rate in the heart of obese mice induced by a single session of exhaustive exercise, upregulated the protein expression of PINK1, SIRT1, PGC-1α, Mfn1, and Drp1, decreased the levels of p62 and Cyt-c, promoted mitophagy, mitochondrial biogenesis, and mitochondrial fission/fusion homeostasis. Then, it also downregulated the levels of BAX protein, increased Bcl-2 and Bcl-2/BAX levels, inhibited cardiac apoptosis, and attenuated single bout of exhaustive exercise-induced cardiac functional impairment (Dun et al., 2023). Aerobic exercise for 8 weeks significantly increased the protein levels of Opa1 and Mfn1, decreased the protein levels of Fis1, Pink, Parkin, and LC3 II, decreased mitochondrial H_2_O_2_ release and mitochondrial O_2_ respiration, and increased mitochondrial Ca2^+^ retention in the hearts of aging rats. It can improve the imbalance of cardiac mitochondrial dynamics, excessive mitophagy, and mitochondrial dysfunction caused by aging, reduce the protein levels of Bax and cleaved-caspase-3, increase the expression of Bcl-2 protein, inhibit myocardial cell apoptosis, and improve mitochondrial and cardiac function in aging rats (No et al., 2020). Four weeks of aerobic exercise significantly increased the expression of PGC-1α, FNDC5, and Irisin, upregulated the expression of Drp1, Parkin, and LC3B, and decreased the level of Fis1 in the skeletal muscle of aging critical limb ischemia (CLI) mice, which subsequently enhanced mitochondrial fission and mitophagy, decreased the number of Tunnel-positive cells, increased ischemic limb mitochondrial MMP and total ATP production, inhibited skeletal muscle apoptosis, and restored limb function and tissue injury in CLI mice. Cellular experiments further confirmed that electrical stimulation significantly upregulated PGC-1α, FNDC5, and Irisin expression in hypoxia and nutrient deprivation-treated adult myocytes, which subsequently upregulated Drp1, Parkin, PINK1, and LC3B protein levels, whereas PGC1a/FNDC5/Irisin knockdown downregulated PINK1, Parkin, DRP1, and LC3B mRNA levels, suggesting that exercise could enhance mitochondrial fission and mitophagy through the PGC1a/FNDC5/irisin pathway and restore muscle damage in aged CLI mice (He W. et al., 2020). In summary, exercise can inhibit apoptosis and reduce metabolic disease risk factors by modulating mitophagy.

#### 5.2.3 Exercise regulates mitophagy to inhibit inflammatory response

Inflammatory responses are closely associated with the development of metabolic diseases (Pirih et al., 2000; Zhang YL. et al., 2022). It was shown that 8 weeks of swimming exercise significantly upregulated Drp1, OPA1, and Mfn2 expression, restored hepatic mitochondrial fusion/fission homeostasis, and upregulated P-AMPK/AMPK, SIRT1, and PGC-1α and their target genes NRF1, NRF2, and TFAM expression. Moreover, it activated the AMPK/SIRT1/PGC-1α pathway, thereby promoting mitochondrial biogenesis in NAFLD zebrafish, while upregulating the levels of PINK1 and Parkin proteins, decreasing the expression of p62, and promoting the level of mitophagy, which then significantly reduced the level of collagen type Ⅰ α1 chain (COL1A1), α-actinin-2 (ACTA2), and IL-β expression in NAFLD zebrafish liver, and increased the expression of anti-inflammatory cytokine IL-10. Further, this attenuated liver inflammation and slowed down the progression of fibrosis in NAFLD zebrafish (Zou et al., 2023). Aerobic exercise for 4 weeks significantly upregulated the expression of FUNDC1 in coronary artery tissues of LAD-induced aging MI/R-injured mice and downregulated the expression of IL-6, IL-8, TNF-α, VCAM-1, E-selectin, and ICAM-1 in left ventricular tissues, which significantly enhanced myocardial glucose uptake, inhibited inflammatory response and neutrophil migration, and attenuated cardiac injury. Further studies confirmed that aerobic exercise significantly upregulated the expression of PPAR-γ in cardiac microvascular ECs of aging MI/R-injured mice, which upregulated the expression of FUNDC1 and, in turn, upregulated the expression of LC3-II proteins and downregulated the level of p62 proteins. This also promoted FUNDC1-mediated mitophagy, reduced senescence-associated β-galactosidase (SA-β-gal) activity, and downregulated the protein expression of p21 and p53, preventing the abnormal migration and proliferation of cardiac microvascular ECs, inflammatory response, and endothelial nitric oxide synthase (eNOS) activation and attenuating cardiac injury (Ma L. et al., 2023). Aerobic exercise for 16 weeks significantly downregulated GRP78 and p-PERK levels in plasma and cardiac tissues of rats injected with deoxycorticosterone acetate (DOCA)-salt-induced hypertension, increased MDA, CAT, SOD1, SOD2, glutathione S-transferase class μ (GSTμ), GPX, NRF2, and PINK1 levels, and significantly decreased NLRP3, caspase-1, and IL-1β expression. Moreover, it enhanced Bcl-2 expression, attenuated p38 and JNK phosphorylation, and improved cardiac function in hypertensive rats, suggesting that exercise may play a protective role against hypertension-induced cardiac dysfunction by regulating cellular stress responses (OS, ERS, mitophagy, inflammatory response, and mitotic activation) (Bal et al., 2022). In summary, exercise may exert metabolic protection by inhibiting the infiltration of inflammatory cytokines in damaged tissues through the regulation of mitophagy.

#### 5.2.4 Exercise-mediated exerkines regulate mitophagy

Exercise stimulates the release of specific endogenous cytokines, peptides, nucleic acids, and metabolites from skeletal muscle and other endocrine organs, collectively called exerkines, which have a crucial regulatory role in maintaining cellular functional homeostasis and quality control (Pedersen et al., 2004; Safdar et al., 2016). A study showed that High-intensity interval exercise significantly increased the p-AMPK/AMPK and p-AMPK/β-actin ratios and the levels of P70S6K, SIRT3, and PGC-1α proteins in the flounder muscle of aged rats, activated the SIRT3/PGC-1α and AMPK signaling pathways, and significantly upregulated Beclin-1, succinate dehydrogenase complex subunit A (SDHA), PINK1, Parkin and SOD2 protein expression and LC3-II/LC3-I ratio, and downregulated Fis1, Drp1 and p62 protein levels, increased IL-15 and OPA1 mRNA expression and mitoSC content, promoted mitophagy, mitochondrial dynamics and mitochondrial biogenesis, and improved mitochondrial function in aged rat flounder muscle (Han C. et al., 2022). Aerobic exercise for 12 weeks significantly upregulated SIRT1 expression in the hippocampus of APP/PS1 transgenic mice, activated the SIRT1-FOXO1/3 signaling pathway, increased the expression of Parkin and LC3-II/Ⅰ proteins, downregulated the levels of PINK1 and P62 proteins, facilitated the degree of co-localization of TOMM20 and LAMP1, and promoted mitophagy, which, in turn, significantly reduced the Aβ plaque load and Aβ_40_ and Aβ_42_ levels, and improved learning and memory ability in APP/PS1 transgenic mice, suggesting that the SIRT1-FOXO1/3 signaling pathway is a potential target for exercise-enhanced mitophagy (Zhao N. et al., 2023). Three weeks of moderate-intensity aerobic exercise significantly increased myocardial FNDC5 expression and upregulated serum Irisin expression in irradiated X-ray-induced radiation-induced heart disease (RIHD) mice, significantly upregulated myocardial Drp1, PINK1, and LC3B expression in RIHD mice, promoted mitochondrial fission and mitophagy, and consequently increased mitochondrial protein and ATP content, decreased myocardial inflammatory cytokines infiltration and myocardial fibrosis area, upregulated LVEF, left ventricular end-diastolic dimension (LVEDD), and systolic left ventricular dimension (SLVD), and increased RIHD mice body weight, grip strength and aerobic fitness. This suggests that aerobic exercise may promote mitochondrial fission and mitophagy through the FNDC5/Irisin signaling pathway, thereby restoring cardiac function and aerobic fitness in RIHD mice (He W. et al., 2021). In summary, exercise mediates the regulation of mitophagy by exerkines and delays the development of metabolic diseases.

## 6 Conclusion and perspectives

Mitophagy is an essential regulatory mechanism that ensures the homeostasis of mitochondrial number and mass and maintains cell survival. It plays a crucial role in metabolic diseases such as diabetic complications, CVDs, NDDs, and MAFLDs. Targeted improvement of mitophagy abnormality may become a novel strategy to prevent and treat metabolic diseases in the future. Exercise, as an important non-pharmacological intervention for disease prevention and rehabilitation, can regulate the level of mitophagy and can intervene in the pathological process of metabolic diseases by regulating mitophagy to inhibit OS, apoptosis, and inflammatory response.

However, there is a lack of systematic research on the optimal exercise modality and exercise program to regulate mitophagy, and the specific mechanism of mitophagy to improve metabolic diseases and its exercise intervention has not been fully elucidated. The exploration and improvement of these issues in the future may provide a new perspective for research related to the regulation of mitophagy to improve metabolic diseases through exercise.
